# Retracted: Caveolin and MAP kinase interaction in angiotensin II preconditioning of the myocardium

**DOI:** 10.1111/j.1582-4934.2012.01619.x

**Published:** 2012-09-26

**Authors:** Manika Das, Samarjit Das, Dipak K Das

**Affiliations:** Cardiovascular Research Center, University of Connecticut School of MedicineFarmington, USA

The following article from *Journal of Cellular and Molecular Medicine* (volume 11, issue 4, 2007, pages: 788–97), ‘Caveolin and MAP kinase interaction in angiotensin II preconditioning of the myocardium' by Manika Das, Samarjit Das and Dipak K Das, published online on 24th June 2007 in Wiley Online Library (http://onlinelibrary.wiley.com), has been retracted by agreement between the authors, the journal Editor-in-Chief, Professor LM Popescu, and Blackwell Publishing Ltd. The retraction has been agreed due to findings of data fabrication in the western blot data in Figure 2a in an investigation into the work of Dipak K. Das by the University of Connecticut Health Center.
